# The Relationship Between Trace Elements and Depression

**DOI:** 10.3390/nu18030484

**Published:** 2026-02-01

**Authors:** Yuanjian Zhong, Yuxiang Nie, Yuanhui Mao, Yinting Liu, Tong Zou, Xiayun Liao, Lichun Zhao

**Affiliations:** 1 College of Pharmacy, Guangxi University of Chinese Medicine, No.13, Wuhe Avenue, Qingxiu District, Nanning 530200, China; zhongyj@gxtcmu.edu.cn (Y.Z.);; 2 Department of Pharmacy, Guangxi Tourism Development Group Guangxi Free Trade Zone Hospital Management Co., Ltd., No. 15 Xinliang Road, Liangqing District, Nanning 530200, China; 3 Research Center of Trace Elements and Health Development, Guizhou University of Traditional Chinese Medicine, 4, Dongqing Road, Huaxi District, Guiyang 550025, China

**Keywords:** depression, trace elements, network pharmacology, signaling pathways, research progress

## Abstract

Trace elements are widely involved in fundamental physiological processes, including enzymatic reactions, neurotransmitter metabolism, and redox homeostasis, and their balanced regulation plays an important role in maintaining normal brain development and neurological function. Depression is a complex psychiatric disorder characterized primarily by mood disturbances, with its onset and progression arising from long-term interactions among genetic susceptibility, neurobiological alterations, and environmental factors. A substantial body of epidemiological and clinical evidence indicates that dysregulation of trace elements—such as zinc, selenium, iron, and magnesium—is closely associated with the risk of depression and the severity of depressive symptoms. Mechanistic studies further demonstrate that trace elements influence depression-related pathophysiology through multi-target and multi-pathway mechanisms, including modulation of monoaminergic neurotransmission, neuroinflammation, oxidative stress, mitochondrial energy metabolism, and hypothalamic–pituitary–adrenal axis function. Network pharmacology analyses have additionally identified systemic hub targets, such as albumin (ALB), insulin (INS), and TP53, as well as key pathways including calcium signaling, neuroactive ligand–receptor interactions, and the HIF-1 signaling pathway. These findings suggest that trace elements may regulate depression-related pathological processes through coordinated network-level effects. Collectively, these integrative insights provide a theoretical basis for the application of trace elements in depression risk assessment, the development of precision intervention strategies, and future mechanistic investigations.

## 1. Introduction

Major Depressive Disorder (MDD) is a chronic psychiatric condition characterized by persistent low mood, anhedonia, cognitive impairment, and disturbances in biological rhythms. Its onset and progression involve complex interactions among genetic susceptibility, environmental stressors, and multilevel neurobiological abnormalities [1]. According to the Global Burden of Disease (GBD) 2021 estimates, approximately 332 million individuals worldwide were living with depressive disorders in 2021, with about 357 million new cases reported during the same year, accounting for more than 56.3 million disability-adjusted life years (DALYs). Although trends in incidence, prevalence, and DALY rates have shown a modest decline, the absolute number of cases and age-standardized rates of depression continue to increase globally [2]. Data from the United States indicate that the annual prevalence of major depressive episodes among adults in 2022 was approximately 7.0% in men and 10.4% in women [3]. Similarly, the latest GBD 2021 analysis in China revealed that the number of individuals with depression increased from 34.4 million in 1990 to 53.1 million in 2021, with a more pronounced rise observed during the COVID-19 pandemic [4]. In addition, a national survey conducted in 2023 reported a detection rate of depressive symptoms as high as 9.8% among Chinese university students, suggesting a notable upward trend of depressive disorders in younger populations [5]. Depression not only substantially increases the risk of suicide but also leads to long-term impairment of work capacity and considerable socioeconomic burden, making it a major contributor to global health-related loss of quality-adjusted life expectancy [6].

Notably, depression is not a single homogeneous disorder but rather a spectrum of affective syndromes with marked clinical and biological heterogeneity [7,8]. Based on disease course, clinical presentation, and the presence or absence of manic or hypomanic episodes, depressive disorders can be broadly classified into unipolar depression, bipolar depression, and several subtypes with specific triggers or circadian features, such as seasonal affective disorder and postpartum depression [9,10,11,12,13]. Distinct depressive subtypes differ in neurotransmitter regulation, endocrine rhythmicity, inflammatory responses, and treatment responsiveness. This heterogeneity is widely regarded as a major factor contributing to variable therapeutic outcomes and the high recurrence rate observed in clinical practice [14,15]. Therefore, elucidating the multidimensional mechanistic basis of depression is essential for the development of precision-oriented interventions. At present, the pathophysiological mechanisms underlying depression remain incompletely understood. Current evidence supports the involvement of multiple interrelated biological pathways, including dysregulation of monoaminergic and glutamatergic neurotransmission, reduced expression of neurotrophic factors such as brain-derived neurotrophic factor (BDNF), impaired neuroplasticity and neurogenesis, aberrant glutamate–*N*-methyl-D-aspartate receptor (NMDAR) signaling, chronic low-grade inflammation, oxidative stress, mitochondrial dysfunction, vascular pathology, as well as interactions between genetic and environmental factors [16,17,18,19,20,21,22,23,24]. Clinically prescribed antidepressants primarily include selective serotonin reuptake inhibitors (SSRIs; e.g., fluoxetine and sertraline), serotonin–norepinephrine reuptake inhibitors (SNRIs; e.g., venlafaxine and duloxetine), tricyclic antidepressants (TCAs), and atypical antidepressants such as bupropion and mirtazapine. These agents exert their antidepressant effects mainly by inhibiting monoamine reuptake, modulating receptor sensitivity, enhancing synaptic plasticity, or rapidly regulating NMDAR signaling. However, their clinical utility is often limited by delayed onset of action, substantial interindividual variability in efficacy, and adverse effects including gastrointestinal discomfort, insomnia, sexual dysfunction, and cardiovascular complications [25,26]. In recent years, non-competitive NMDAR antagonists such as ketamine have demonstrated rapid and robust antidepressant effects in treatment-resistant depression by modulating glutamatergic transmission and promoting synaptic plasticity. Nevertheless, concerns regarding psychotomimetic symptoms, transient hypertension, and abuse potential continue to restrict their widespread clinical application [27,28,29].

Given the high complexity of the pathophysiology of depression and the limitations of current therapeutic strategies, increasing attention has been directed toward the potential role of trace element homeostasis in the onset, progression, and intervention of depressive disorders. Trace elements are defined as elements present in the human body at concentrations ranging from approximately 0.01% to 0.005% of body weight, including zinc (Zn), iron (Fe), selenium (Se), and copper (Cu). These elements are involved in multiple neurobiological processes, such as neurotransmitter synthesis, maintenance of synaptic plasticity, regulation of redox homeostasis, and modulation of immune and inflammatory responses. Deficiency or excess of trace elements may alter the structure and function of emotion-related brain regions, including the hippocampus and amygdala, thereby increasing susceptibility to affective disorders [30,31]. For instance, zinc, acting as a ligand of the zinc-sensing receptor GPR39, can activate G protein–mediated signaling pathways and modulate the expression of brain-derived neurotrophic factor (BDNF), which has been implicated in the pathogenesis of depression as well as antidepressant responses [32,33]. Magnesium serves as a physiological blocker of the *N*-methyl-D-aspartate receptor (NMDAR), thereby regulating glutamatergic signaling and participating in the functional modulation of the hypothalamic–pituitary–adrenal (HPA) axis [34,35]. Iron is an essential cofactor for the synthesis of dopamine (DA) and norepinephrine (NE), and iron deficiency may disrupt monoaminergic neurotransmitter metabolism [36]. Selenium is a structural component of several antioxidant enzymes, including glutathione peroxidase (GPx), and alterations in selenium status have been closely associated with depressive disorders [37,38]. Moreover, supplementation with selenium or chromium has been reported to significantly improve depressive symptoms in frail elderly populations [39]. Collectively, these findings suggest that maintenance of trace element homeostasis may modulate neurobiological processes through multiple targets and pathways, providing potential avenues for integrative interventions in depression.

Notably, network pharmacology has emerged as an interdisciplinary research paradigm integrating systems biology, network analysis, and multi-target drug design. This approach emphasizes the investigation of disease mechanisms and therapeutic effects from a holistic network perspective, thereby moving beyond the traditional “single target–single drug–single disease” model [40]. Given that trace elements exert their biological effects through multiple targets and signaling pathways involved in neurotransmission, oxidative stress regulation, and immune–inflammatory responses, their functional characteristics are highly consistent with the systemic and multi-target principles of network pharmacology. Accordingly, network pharmacology provides a suitable theoretical framework and analytical tool for systematically elucidating the molecular mechanisms by which trace elements contribute to disease development and progression [41,42].

Based on this, the present study provides a systematic review of the research progress on trace element homeostasis dysregulation in the onset and progression of depression. It focuses on the potential roles of trace elements in neurobiological regulation and, by integrating network pharmacology approaches, identifies key targets and signaling pathways through which trace elements may exert their effects. This work aims to provide a theoretical basis and research framework for multi-target interventions and precision therapeutic strategies in depression.

## 2. Trace Elements with Potential Beneficial Effects on Depression

### 2.1. Iron (Fe)

Iron is an essential trace element for living organisms and a critical component of hemoglobin, participating in oxygen transport, DNA synthesis, mitochondrial electron transport chain activity, and adenosine triphosphate (ATP) production [43,44,45,46]. The biological functions of iron are largely based on its ability to undergo reversible redox cycling between the Fe^2+^ and Fe^3+^ states. Iron deficiency leads to iron-deficiency anemia, whereas iron overload can catalyze the generation of reactive oxygen species, thereby inducing oxidative stress and resulting in cellular damage or even cell death [47]. In the central nervous system (CNS), iron serves as a key cofactor for dopamine β-hydroxylase (DBH) and tyrosine hydroxylase (TH), enzymes that are essential for the biosynthesis of catecholaminergic neurotransmitters [48,49,50]. Adequate tissue iron levels are critical for the integrity of the dopaminergic system and are closely associated with dopamine D2 receptor expression and dopamine transporter (DAT) density, thereby influencing the regulation and functional activity of dopaminergic neurons [51,52]. In addition, iron-dependent myelination requires the coordinated actions of divalent metal transporter 1 (DMT1) and the iron exporter ferroportin (FPN) to maintain intracellular iron homeostasis in oligodendrocytes, ensuring proper myelin formation and efficient neural signal conduction [53,54]. Through the regulation of monoaminergic neurotransmitter metabolism, including dopamine, norepinephrine, and serotonin, as well as its involvement in myelination and cerebral energy metabolism, iron plays an important role in maintaining synaptic plasticity, neurodevelopment, and the stability of emotion-related neural circuits [55]. Consequently, disruption of iron homeostasis is considered likely to interfere with neural networks involved in emotional regulation, thereby contributing to the onset and progression of depression.

Existing evidence suggests that abnormal brain iron deposition may contribute to the onset and progression of depression and could potentially serve as an imaging biomarker reflecting underlying pathophysiological changes [56,57]. At the central level, quantitative magnetic resonance imaging studies have revealed region-specific alterations in iron content in patients with major depressive disorder (MDD). For instance, bilateral globus pallidus and caudate nucleus exhibit reduced iron levels, whereas the thalamus and putamen show increased iron accumulation, with these changes significantly correlating with local gray matter volume [58]. Similarly, Mendelian randomization analyses further support region-specific potential causal links between brain iron homeostasis and psychiatric disorders: decreased iron levels in the left substantia nigra may elevate depression risk, while increased iron in the putamen is associated with other neuropsychiatric phenotypes [59]. Collectively, these findings indicate that brain iron dysregulation does not occur as a uniform increase or decrease but may influence emotion regulation through specific neural circuits, such as the basal ganglia–thalamus–cortex pathway. Notably, some studies have observed that thalamic iron accumulation is independently associated with depressive symptoms, without a significant correlation with white matter hyperintensity volume [60], further supporting the notion that the impact of brain iron on depression exhibits structural specificity. Such regional differences may arise from variations in iron uptake, storage, and utilization across brain regions, as well as their distinct roles in neurotransmitter synthesis, mitochondrial energy metabolism, and oxidative stress regulation [61,62]. At the population level, multiple epidemiological studies have consistently suggested a potential association between abnormal peripheral iron status and depressive symptoms, although the directionality and population specificity of this relationship exhibit substantial heterogeneity. Survey data indicate that iron deficiency across different life stages is associated with depression, particularly among adults aged ≥ 65 years, in whom iron deficiency frequently co-occurs with depressive symptoms [63]. Among non-pregnant women of reproductive age (WRA, 20–40 years), iron deficiency has been associated with a higher prevalence of depression and increased symptom severity compared with iron-replete women [30]. Moreover, increased iron metabolic demands during pregnancy may elevate the risk of depression in women [64]. An analysis based on national databases has shown that iron deficiency anemia (IDA) is significantly associated with an increased risk of anxiety, depression, sleep disorders, and psychotic disorders and that iron supplementation is associated with a reduced risk of psychiatric disorders in certain populations [65]. However, under specific physiological conditions, improvements in iron status do not always correspond to parallel changes in depression risk. A retrospective cohort study reported no significant difference in the incidence of postpartum depression among women receiving intravenous iron therapy for prenatal anemia [66]. Similarly, another cohort study found no direct association between improved iron levels and postpartum depression, although amelioration of anemia severity in early pregnancy was likely to alleviate the overall burden of postpartum depression [67]. These findings suggest that during pregnancy and the perinatal period, the development of depression may be more strongly influenced by multiple factors, including hormonal fluctuations, inflammatory responses, and psychosocial stressors, thereby limiting the independent effect of iron supplementation.

In addition, inconsistencies among studies may be substantially influenced by methodological differences and confounding factors. A case–control study did not observe a direct association between depression and reduced iron levels but reported a significantly higher rate of iron supplementation in the case group than in controls, suggesting that a history of iron therapy—particularly among women—may introduce notable bias [68]. This observation underscores the need to consider iron supplementation history, especially in female populations, as an important confounding variable when evaluating the relationship between iron status and depression.

In populations with concomitant neurological disorders, the association between iron homeostasis and depression is further modulated by the pathological processes of the underlying diseases. For example, in patients with multiple sclerosis, ferritin deficiency has been associated with more severe depressive symptoms and reduced quality of life [69]. In patients with Parkinson’s disease, depression scores are negatively correlated with serum iron levels and positively correlated with transferrin levels [70,71], indicating that the relationship between iron metabolism abnormalities and depressive symptoms may be further shaped by disease-specific pathology, thereby limiting the generalizability of these findings to the general population. Moreover, sex differences represent an additional modifying factor. Some studies have reported that elevated iron levels are associated with increased depressive symptoms in adult men, whereas no comparable association has been observed in adult women [72]. This discrepancy may be related to differences in hormonal profiles, iron storage patterns, and menstruation-related iron metabolism.

Taken together, existing evidence suggests that iron homeostasis dysregulation does not contribute to depression through a single or linear mechanism. Instead, it is more likely to reflect the pronounced biological heterogeneity of depression through imbalances in central–peripheral iron regulatory networks. The association between iron status and depression appears to be highly dependent on specific populations, physiological stages, and dose ranges within a dynamic homeostatic framework, resulting in seemingly contradictory findings across studies, including the coexistence of deficiency and overload effects, sex- and age-related differences, and U-shaped relationships. This heterogeneity indicates that peripheral iron status not only reflects systemic iron stores but is also strongly influenced by inflammatory burden, stress responses, and metabolic state, and therefore cannot be simply equated with central iron homeostasis. Consequently, current evidence supports viewing iron homeostasis abnormalities as an important biological dimension that modulates depression susceptibility, clinical phenotype, and treatment response, rather than as a universal etiological factor applicable to all patients. Future studies are urgently needed to integrate iron status, physiological stage, sex differences, and iron supplementation history within longitudinal research frameworks to more precisely delineate the temporal and contextual roles of iron homeostasis dysregulation in the onset and progression of depression.

**Figure 1:**
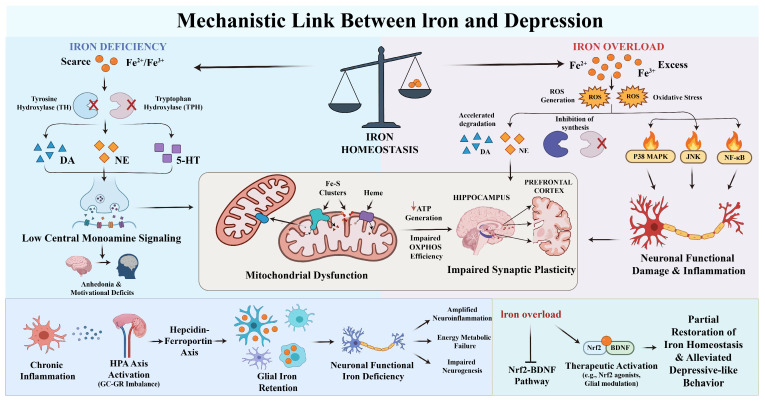
Mechanistic pathways linking iron dysregulation to depression (for illustrative purposes only). Iron deficiency impairs tyrosine and tryptophan hydroxylase activity, reducing dopamine (DA), norepinephrine (NE), and serotonin (5-HT) synthesis, leading to anhedonia and motivational deficits. Iron overload promotes reactive oxygen species (ROS) production, oxidative monoamine degradation, and activates stress- and inflammation-related pathways (p38 MAPK, JNK, NF-κB), causing neuronal dysfunction. As a critical component of mitochondrial Fe–S clusters and heme, iron imbalance disrupts oxidative phosphorylation (OXPHOS) and ATP generation, impairing synaptic plasticity in mood-regulating regions such as the hippocampus and prefrontal cortex. Chronic inflammation and HPA axis hyperactivation alter hepcidin–ferroportin and glucocorticoid receptor (GR) signaling, promoting glial iron accumulation and functional neuronal iron deficiency. Protective pathways including Nrf2–BDNF are suppressed under iron overload, whereas modulation of glial iron metabolism, anti-inflammatory polarization, or Nrf2 activation can restore iron homeostasis and ameliorate depressive-like behaviors. (Arrow definitions: Solid arrows indicate direct regulatory or causal relationships supported by relatively robust experimental evidence; bidirectional arrows represent reciprocal or feedback regulatory processes; T-shaped terminal lines indicate inhibitory or negative regulatory effects.)

At the mechanistic level, iron serves as an essential cofactor for multiple aromatic hydroxylases. Iron deficiency reduces the catalytic activity of tyrosine hydroxylase and tryptophan hydroxylase, thereby suppressing the biosynthesis of dopamine (DA), norepinephrine (NE), and serotonin (5-HT), and weakening central monoaminergic signaling. Chronic impairment of monoamine signaling contributes to core depressive symptoms, including anhedonia, apathy, and reduced motivation and reward responsiveness [73,74,75]. Conversely, iron overload accelerates the autoxidation of catecholamines through oxidative stress, generating neurotoxic compounds, decreasing cerebral DA and 5-HT levels, and inhibiting enzymes such as tyrosine hydroxylase, further impairing neuronal function and neurotransmitter synthesis pathways [76,77]. Iron also acts as a central trace element in cellular energy metabolism and redox reactions, playing an irreplaceable role in maintaining mitochondrial integrity and cerebral energy supply, with mitochondrial dysfunction widely recognized as a pivotal factor in depression pathophysiology [78,79,80]. Mitochondria are the primary site of ATP generation, where oxidative phosphorylation (OXPHOS) sustains the energy demands of neurons and glial cells. Disrupted iron homeostasis reduces mitochondrial iron availability, impairing the function of Fe–S clusters and heme groups within respiratory chain complexes I–IV, resulting in diminished ATP production and energy deficiency. Such deficits particularly compromise synaptic plasticity and network function in hippocampal and prefrontal circuits involved in mood regulation, exacerbating depressive symptoms [61,81,82,83,84]. Iron overload and elevated serum iron induce dysregulation of iron homeostasis via Nrf2 downregulation, accompanied by upregulation of transferrin receptor (TfR) and increased iron deposition. These changes suppress BDNF-mediated synaptic plasticity and disrupt hippocampal and limbic system connectivity, thereby aggravating neural circuit dysfunction and depressive-like behaviors. Activation of Nrf2 can restore iron homeostasis and reduce depression susceptibility [85]. Furthermore, iron overload impairs electron transport chain (ETC) function and activates stress-response signaling pathways, including p38 MAPK, JNK, and NF-κB, via iron-mediated reactive oxygen species (ROS), promoting neuroinflammatory cascades and programmed cell death [86,87,88]. Low-grade, persistent inflammatory signals, particularly IL-6, upregulate hepcidin and promote degradation of ferroportin, leading to intracellular iron sequestration resembling anemia of inflammation or functional iron blockade. Prolonged conditions of reduced bioavailable iron impair mitochondrial function and exacerbate cognitive and emotional dysregulation [89]. Ke et al. further demonstrated that chronic stress persistently activates the hypothalamic–pituitary–adrenal (HPA) axis, shifting iron metabolism from adaptive regulation to pathological imbalance. This manifests as excessive iron retention in specific cell types (e.g., microglia and macrophages) and functional iron deficiency in neurons or oligodendrocytes. Such compartmentalized iron distribution enhances oxidative stress and inflammation, diminishes mitochondrial efficiency, and ultimately impairs synaptic function and neural circuit stability, linking chronic stress to neuronal damage and depression pathogenesis [90,91,92]. Similarly, chronic unpredictable mild stress (CUMS) and brain iron deficiency downregulate glucocorticoid–glucocorticoid receptor (GC–GR) signaling, induce GR dysfunction, weaken hippocampal negative feedback on the HPA axis, and consequently lead to neuronal injury and reduced neurogenesis, promoting depression onset and progression [93]. Targeted interventions at multiple levels demonstrate potential antidepressant effects. Eicosapentaenoic acid (EPA) and docosahexaenoic acid (DHA) increase oligodendrocyte markers Olig2, myelin basic protein (MBP), and ferritin heavy chain (FTH), reduce lipid peroxidation in oligodendrocytes, inhibit astrocyte activation and the LCN2–NLRP3 inflammatory pathway, correct microglial M1/M2 polarization, and alleviate depressive-like behaviors in chronically sleep-deprived mice [94]. At the transcriptional level, Nrf2 signaling is crucial for maintaining iron homeostasis. Pharmacological activation of Nrf2 restores iron balance, prevents BDNF suppression, protects hippocampal and limbic connectivity, and reduces depressive susceptibility [85]. Fumarate derivatives also activate Nrf2, upregulate anti-ferroptotic enzymes in oligodendrocytes and associated tissues, and decrease iron-dependent lipid peroxidation and myelin damage, thereby exerting anti-inflammatory and neuroprotective effects [95]. Collectively, dysregulated iron homeostasis is not a single causal factor in depression but interacts with monoamine metabolism, neuroinflammation, glial dysfunction, gut microbiota and its metabolites, and epigenetic alterations, amplifying oxidative stress and neural circuit damage and forming a highly complex and dynamically plastic pathological network (Figure 1).

### 2.2. Zinc (Zn)

Zinc, as an essential trace element, plays a critical role in brain development, structural maintenance, and mood regulation [96]. Zinc is enriched in brain regions closely associated with emotion regulation, including the hippocampus, prefrontal cortex, amygdala, and olfactory bulb, and participates in neurotransmitter modulation, synaptic plasticity, redox homeostasis, and neuroinflammatory responses [97,98,99]. Dysregulated zinc homeostasis may therefore interfere with neural circuit function through multiple targets, increasing the risk of mood disorders [100]. Clinical studies indicate that patients with treatment-resistant depression exhibit lower serum Zn^2+^ levels compared to non-treatment-resistant patients, suggesting that zinc deficiency may correlate with depression severity and treatment responsiveness [101]. Zinc deficiency typically results from insufficient dietary intake, increased demand, or accelerated metabolism associated with chronic illness, whereas adequate zinc intake is inversely associated with depression risk [102,103]. In postmenopausal women, higher serum zinc levels are linked to less severe depressive symptoms, and zinc supplementation may serve as a therapeutic strategy for zinc-deficient women in this population [104,105,106]. In contrast, the association between zinc levels and depressive symptoms in men appears to be relatively inconsistent [107,108], suggesting that sex differences may shape the phenotypic variability of the zinc–mood relationship through combined effects of endocrine regulation, baseline zinc reserves, and metabolic adaptability. At the interventional level, a randomized, double-blind, placebo-controlled trial demonstrated that zinc monotherapy increased serum brain-derived neurotrophic factor (BDNF) levels and alleviated depressive symptoms in overweight or obese subjects [109]. Systematic reviews and meta-analyses of clinical trials further suggest that zinc supplementation may exert synergistic effects when combined with antidepressant therapy, contributing to symptom improvement [110]. Notably, one randomized controlled study reported that zinc supplementation significantly reduced relapse risk in patients with opioid use disorder while simultaneously improving depressive, anxiety, and stress-related symptoms, implying that zinc may influence shared pathological mechanisms of mood disorders and addiction through modulation of neurotransmitter homeostasis, neuroinflammation, and stress-related pathways [111]. Additionally, meta-analyses indicate a consistent inverse association between dietary zinc and iron intake and depression risk, with zinc showing higher consistency across multiple subgroups and sensitivity analyses [112]. Recent systematic reviews further confirm that higher zinc intake or supplementation is significantly associated with reduced depression risk and symptom improvement, with some studies demonstrating more pronounced effects of zinc monotherapy, supporting its role in modulating neurotransmitter function, antioxidant defense, and neuroinflammatory responses in depression onset and intervention [113].

Although the overall body of evidence supports an association between zinc homeostasis dysregulation and the onset, severity, and treatment response of depression, substantial heterogeneity and methodological limitations remain across existing studies. Considerable variability exists in zinc exposure assessment methods (e.g., dietary intake, serum zinc, or intracellular zinc levels), study designs, and population characteristics, which limits the comparability of findings. Moreover, the predominance of cross-sectional studies hampers the establishment of causal relationships. In addition, the biological effects of zinc may exhibit dose-dependent or even nonlinear characteristics, whereas the potential risks associated with excessive supplementation have not yet been systematically evaluated. Overall, zinc should be regarded as an adjunctive intervention with potential target-specific value rather than a universal antidepressant treatment, and its clinical application should be grounded in individualized assessment and the continued refinement of evidence-based support.

The potential mechanisms by which zinc exerts antidepressant effects involve multi-level neuroendocrine and neuroimmune regulatory networks, including hypothalamic–pituitary–adrenal (HPA) axis modulation, excitatory/inhibitory neurotransmitter balance, zinc homeostasis and receptor-mediated signaling, monoaminergic system regulation, and suppression of neuroinflammatory responses [114,115]. In stress-related pathways, reduced serum zinc is often associated with elevated corticosterone levels, which may induce chronic HPA axis hyperactivation, thereby disrupting serotonergic (5-HT) and noradrenergic (NA) circuit homeostasis and enhancing glucocorticoid-mediated negative affect [116]. Experimental studies indicate that corticosterone stimulates hippocampal zincergic neurons, promoting Zn^2+^ release, and excessive free zinc may trigger hippocampal dysfunction and depressive-like behavior [117]. At the neurotransmitter regulation level, zinc modulates emotion through multiple excitatory and inhibitory receptors, including *N*-methyl-D-aspartate (NMDA) receptors, α-amino-3-hydroxy-5-methyl-4-isoxazolepropionic acid (AMPA) receptors, metabotropic glutamate receptors (mGluRs), and γ-aminobutyric acid (GABA) receptors [118,119]. Zinc deficiency can increase reactive oxygen species (ROS) production and overactivate NMDA receptors, leading to excitotoxicity and accelerated serotonin depletion, thereby exacerbating depressive phenotypes [105,120]. Neuropeptide Y (NPY), an important stress-buffering factor widely distributed in depression-associated regions such as the hypothalamus, hippocampus, and amygdala, regulates feeding behavior and HPA axis function [121]. Zinc deficiency significantly upregulates hypothalamic NPY and its mRNA expression, suggesting that zinc dysregulation may affect emotion and energy metabolism via abnormal NPY signaling [122]. At the cellular level, altered expression of zinc transporters is observed in the prefrontal cortex of depressed patients, with elevated ZnT1, ZnT4, and ZnT5 levels and decreased synaptic vesicle-associated ZnT3, which correlates with suicidal tendencies, indicating that disrupted zinc transmembrane transport may contribute to depression neuropathology [123]. Zinc also exerts neuromodulatory effects through activation of G protein-coupled receptor 39 (GPR39), broadly expressed in the prefrontal cortex, amygdala, and hippocampus, with activation associated with antidepressant-like effects [124]. Zinc deficiency reduces brain NE levels and downregulates the norepinephrine transporter (NET), impairing synaptic NE clearance and elevating plasma MHPG, thereby inducing anxiety- or depression-like behavior [125]. Notably, zinc enhances the rapid antidepressant effects of imipramine via a protein kinase A (PKA)-dependent mTOR/CREB signaling pathway [126]. Combined zinc and fluoxetine treatment reverses hippocampal histone deacetylase (HDAC) dysregulation in chronic restraint stress (CRS) models, suggesting a potential epigenetic mechanism by which zinc enhances conventional antidepressant efficacy [127]. At the neuroimmune level, neuroinflammation is a core pathological mechanism of depression, impairing hippocampal glucocorticoid receptor function and disrupting stress feedback regulation [128,129]. Both in vitro and in vivo evidence indicate that zinc inhibits the P2X7–NLRP3 inflammasome signaling axis, suppresses microglial M1 polarization, and promotes M2 polarization, thereby attenuating central nervous system inflammation and exerting potential antidepressant effects [130].

**Figure 2:**
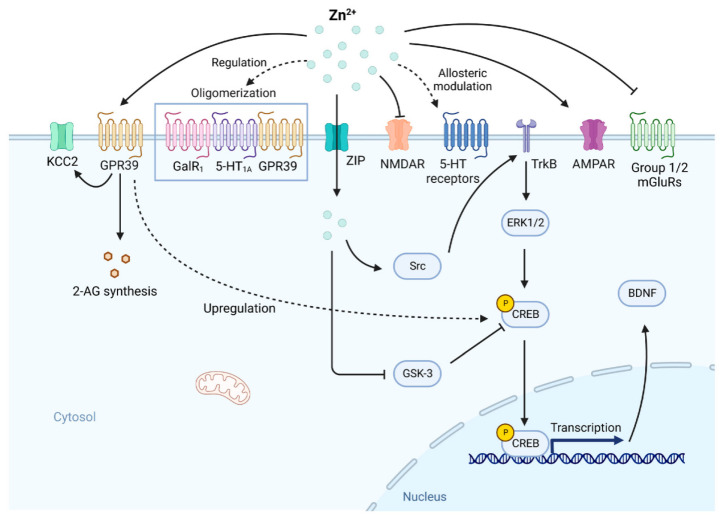
The involvement of Zn^2+^ deficiency in the abnormal neurotransmission and neurotropic signaling in depression (for illustrative purposes only). In normal conditions, the activities of glutamatergic receptors including NMDAR and α-amino-3-hydroxy-5-methyl-4-isoxazole propionic acid receptor (AMPAR), need to be regulated by Zn^2+^. The activation of GPR39 by Zn^2+^ is essential for endocannabinoid 2-arachidonoylglycerol (2-AG) synthesis and the upregulation of potassium chloride co-transporter 2 (KCC2). GPR39 activation can upregulate cAMP response element binding protein (CREB) expression, thus increasing BDNF expression. Zn^2+^ is involved in the allosteric modulation of 5-HT receptors and the regulation of the oligomerization state between GalR1-5-HTA1-GPR39. Zn^2+^ is also an antagonist of group I and group II mGluRs, which are linked to depression. Intracellular Zn^2+^ participates in the modulation of neurotropic signaling, which is crucial in depression. Zn^2+^ can transactivate TrkB and downstream ERK1/2 and CREB via Src family kinase and, on the other hand, inhibit glycogen synthase kinase-3 (GSK-3), which inhibits CREB activity, leading to the expression of BDNF. Under Zn^2+^ deficiency, these activities associated with depression are disrupted, contributing to the development of depression [131]. Arrow definitions: Solid arrows indicate direct regulatory or causal relationships supported by relatively robust experimental evidence; dashed arrows denote putative regulatory associations inferred from correlational studies or systems biology analyses that require further mechanistic validation; T-shaped terminal lines indicate inhibitory or negative regulatory effects. Reproduced from Wang et al., [131], licensed under CC BY 4.0 (https://creativecommons.org/licenses/by/4.0/; accessed on 6 December 2025).

Collectively, current evidence indicates that zinc exerts context-dependent, multi-level regulatory effects in depression onset and intervention. Its biological effects are not mediated by a single signaling pathway but are embedded within a highly coupled network of stress responses, neurotransmitter regulation, immune-inflammatory signaling, and energy metabolism. Dysregulated zinc homeostasis can synergistically affect neural plasticity and mood regulation via HPA axis hyperactivation, excitatory/inhibitory neurotransmitter imbalance, disrupted zinc transport, and amplified neuroinflammatory signaling (Figure 2). This “zinc-centered” network model helps explain observed sex differences, population heterogeneity, and dose-dependent effects in prior studies. Overall, zinc provides an important yet incompletely understood perspective for understanding the systemic pathophysiology of depression, and its potential clinical value awaits further mechanistic validation and stratified intervention studies.

### 2.3. Selenium (Se)

Selenium is an essential trace quasi-metal element that is crucial for human health, and its homeostasis is fundamental for normal physiological function [132]. The total selenium content in the human body is approximately 20 mg, primarily existing in the form of selenoproteins, which play critical roles in antioxidant defense, redox regulation, and energy metabolism, thereby maintaining cellular homeostasis and tissue function [133]. Epidemiological studies indicate that adequate selenium intake is associated with reduced risk for various conditions, including certain skin disorders, digestive system cancers, and depression [134,135]. In the context of mental health, previous studies suggest that moderate dietary selenium intake may be associated with a lower risk of depression and may improve neurological outcomes in post-stroke patients [103]. Population stratification analyses suggest that maintaining serum selenium levels around 82–85 μg/L in young adults is significantly associated with a reduced risk of depressive symptoms, implying the existence of a relatively narrow “optimal physiological range” for selenium [136]. Notably, the biological effects of selenium exhibit marked dose-dependency and bidirectionality. Several studies report that high selenium exposure or chronic overconsumption may paradoxically increase the risk of depression [137]. A nested case-control study in an Australian cohort indicated that low selenium intake significantly increased the risk of incident major depressive disorder (MDD), suggesting that selenium, as an antioxidant and redox regulator, may confer protective effects in depression pathophysiology [138]. Systematic reviews and meta-analyses encompassing approximately 20 observational and interventional studies have found that, overall, serum selenium levels are not significantly correlated with depression scores, and no substantial differences were observed between depressed patients and healthy controls. However, higher selenium intake, particularly in relation to postpartum depression, was negatively associated with risk, and selenium supplementation significantly alleviated depressive symptoms. These findings suggest that selenium intake may better reflect its biological effects in depression than serum concentration [139]. Similarly, a study of Brazilian farmers reported that high selenium intake was associated with lower depression prevalence even after adjusting for sociodemographic factors, lifestyle, and pesticide exposure [140]. However, some systematic reviews have indicated that, owing to substantial heterogeneity in study design, selenium assessment methods, and population backgrounds, the current evidence is insufficient to support a generalized and stable positive association between selenium status and depression, highlighting the need for larger-scale epidemiological studies with more rigorous designs for further validation [141]. In addition, under specific clinical conditions, such as in patients with chronic kidney disease, lower serum selenium levels have been associated with higher depression scores, suggesting that selenium insufficiency may exert more pronounced adverse effects on mood disorders in populations exposed to high oxidative stress or increased disease burden [142].

Taken together, evidence from basic research, epidemiological studies, and clinical intervention trials suggests that the relationship between selenium and depression does not follow a simple linear “deficiency–supplementation” pattern but rather conforms to a homeostatic regulatory model. The biological effects of selenium appear to be highly dependent on the individual physiological range, disease context, and long-term exposure levels. Discrepancies across studies may arise from the combined influence of multiple modifying factors, including differences in age, sex, and physiological stage; variations in underlying disease status or inflammatory burden; limitations of selenium exposure assessment methods (e.g., dietary intake versus single-time-point serum measurements) in reflecting long-term homeostasis; and the inherent constraints of cross-sectional studies in causal inference. Notably, in populations with high oxidative stress or chronic diseases, the potential adverse effects associated with selenium insufficiency are more likely to manifest and be amplified, whereas in the general population, such effects tend to be relatively modest and may even be difficult to detect at a statistical level. Therefore, from the perspective of systems biology and homeostatic regulation, future research should move beyond correlation analyses based on single exposure indicators and, within longitudinal study frameworks, integrate selenium intake levels, endogenous homeostatic regulatory capacity, biomarkers of inflammation and oxidative stress, and central nervous system functional outcomes. Such efforts are needed to more precisely define the susceptible populations and critical windows in which selenium homeostasis dysregulation contributes to the onset and progression of depression, thereby facilitating a shift from population-level associative descriptions toward a more mechanism-oriented and refined understanding.

Mechanistically, selenium contributes to the formation of key antioxidant enzymes, including glutathione peroxidases and thioredoxin reductases, thereby enhancing cellular defense against oxidative damage and buffering the elevated oxidative stress and reactive oxygen species commonly observed in depression [143,144]. Selenium deficiency impairs the function of these antioxidant enzymes, exacerbates oxidative damage and inflammatory responses, and may thereby facilitate the onset of depressive-like behaviors [115]. Selenium is also an essential component of various selenoproteins, including selenoprotein-binding proteins and selenoenzymes. Animal studies have demonstrated that the expression of selenoprotein binding protein 1 (SELENBP1) is reduced in chronic unpredictable mild stress (CUMS)-induced depression models and that genetic deletion of SELENBP1 exacerbates inflammation, oxidative stress, and impairs neurogenesis, further indicating a molecular link between selenium-related proteins and depressive phenotypes [145]. Moreover, organoselenium compounds, such as 1-methyl-3-(phenylselanyl)-1H-indole (MFSeI), have been shown to reverse depressive- and anxiety-like behaviors induced by intracerebroventricular streptozotocin injection in mice [146]. By mitigating oxidative stress and inflammatory responses, selenium may exert beneficial effects on psychopathology and other diseases associated with oxidative and inflammatory dysregulation [147].

Overall, although current evidence on the causal relationship between selenium and depression remains inconclusive, the interplay between trace selenium levels and oxidative/inflammatory burden, together with selenium’s regulation of antioxidant systems and neurotransmitter homeostasis, provides a biological basis for its involvement in depression pathophysiology. Selenium may influence depression development via multiple mechanisms, including modulation of antioxidant defenses, inflammatory pathways, and neurotransmitter balance. However, due to heterogeneity in research methodology, selenium assessment metrics, and depression diagnostic criteria, further large-scale, rigorously designed epidemiological studies and mechanistic experiments are required to clarify the specific role and therapeutic potential of selenium in depression.

### 2.4. Iodine (I)

Iodine, as the sole essential trace element required for thyroid hormone synthesis, profoundly influences mood and neuropsychiatric status through regulation of the hypothalamic–pituitary–thyroid (HPT) axis [148,149]. Adequate iodine intake is fundamental for mammalian survival, as it constitutes a critical component of thyroid hormones, which are essential for growth and development, protein metabolism, maintenance of basal metabolic rate, carbohydrate and lipid metabolism, water–salt balance, and regulation of oxidative stress [150,151,152]. In younger populations, low habitual iodine intake or iodine deficiency increases the risk of perinatal mood disturbances and depression, with anhedonia as a prominent symptom and a predisposition to anorexia [153,154]. A prospective study based on the Norwegian Mother, Father and Child Cohort further demonstrated that insufficient iodine intake from habitual diets during pregnancy was independently associated with higher depression scores during pregnancy and the postpartum period, and this association remained robust after adjustment for potential confounding factors [155]. These findings suggest that during physiological stages characterized by markedly increased thyroid hormone demand, iodine insufficiency is more likely to disrupt hypothalamic–pituitary–thyroid (HPT) axis homeostasis, thereby amplifying its adverse effects on mood regulation. Meanwhile, a cross-sectional analysis of NHANES data from 2007 to 2018 revealed a nonlinear dose–response relationship between urinary iodine concentration (UIC) and depressive symptoms. Specifically, UIC levels above the normal range were associated with a significantly increased risk of depression, while in women, low UIC was also linked to a higher risk of depressive symptoms, with this association being particularly evident among individuals aged 40–59 years [156]. Consistently, a multiethnic cohort study in the United States reported that both iodine intake below and above recommended levels during pregnancy was positively associated with postpartum anhedonia and depressive symptoms, further supporting a U-shaped relationship between iodine status and mood outcomes [157]. In addition, age-related differences have been observed across studies. Among individuals aged 46 years and older, severe depressive symptoms were significantly associated with abnormal UIC levels [158], whereas in adolescent populations, some studies did not detect significant differences in UIC between patients with major depressive disorder and healthy controls [159]. These findings suggest that age-related variations in endocrine sensitivity, stress exposure, and psychosocial factors may modulate the strength of the association between iodine homeostasis and emotional outcomes.

Overall, current epidemiological evidence indicates a significant but nonlinear association between iodine nutritional status and depressive symptoms, with both iodine deficiency and excessive exposure potentially increasing depression risk in specific populations. Inconsistencies among study findings may be attributable to population heterogeneity, differences in endocrine background, and the limited capacity of short-term exposure indicators, such as UIC, to reflect long-term iodine homeostasis. Owing to the predominance of cross-sectional designs and variability in iodine intake assessment methods, direct causal evidence supporting iodine-mediated mood alterations through thyroid dysfunction remains limited. Future studies based on longitudinal cohorts and randomized controlled trials are warranted to systematically integrate iodine intake, thyroid function, and emotional outcome measures, thereby clarifying the critical windows, susceptible populations, and potential pathogenic mechanisms through which iodine homeostasis dysregulation contributes to depression.

## 3. Potentially Harmful Trace Elements in Depression

### 3.1. Lead (Pb)

Heavy metal toxicity represents a global public health crisis, and lead (Pb) is widely recognized as a neurotoxic environmental contaminant associated with mood disorders. Excessive lead exposure has been reported to exacerbate depressive symptoms and other health outcomes [160]. Compared with non-miners, miners exhibit elevated blood concentrations of heavy metals, particularly lead [161]. Cross-sectional analysis of the US NHANES dataset evaluated the relationship between adult blood lead levels and depressive symptom scores. While associations at environmental exposure levels were not always statistically significant, higher exposure categories showed a modestly increased risk of depression, suggesting that environmental lead exposure may have subtle effects on mental health [162]. In occupational cohorts with high lead exposure, blood lead concentrations were positively associated with depression, anxiety, and anger, indicating that elevated lead levels may predict increased risk of mood disturbances in heavily exposed populations [163]. Lead can cross the blood–brain barrier, accumulate in neural tissue, and disrupt neurotransmitter release, ion channel function, and synaptic integrity, thereby directly perturbing neuronal signaling [164]. Importantly, lead exposure strongly induces oxidative stress, characterized by increased reactive oxygen species (ROS) generation, impaired antioxidant defenses (including glutathione and related enzymes), lipid peroxidation, and DNA damage. These processes are widely acknowledged as central features of lead-induced neurotoxicity [165,166]. Persistent oxidative stress can activate inflammatory signaling pathways and impair synaptic plasticity, which are critical pathological mechanisms underlying depression and other mood disorders. Chronic lead exposure induces anxiety- and depressive-like behaviors in mice, potentially mediated by hyperexcitation of excitatory neurons in the ventral dentate gyrus (vDG) subregion of the hippocampus [167]. Lead exposure also promotes depressive- and anxiety-like phenotypes via oxidative stress and neuroinflammation, which can be attenuated through modulation of BDNF signaling [168]. Mechanistically, environmental lead exposure can directly bind to hydroxylation sites on hypoxia-inducible factor-1α (HIF-1α) to induce VDAC1 transcription, thereby promoting astrocytic ferroptosis and mitochondrial dysfunction, contributing to depressive-like behaviors [169]. Furthermore, lead exposure alters gut microbiota composition and the gut–brain axis (GBA), affecting serotonergic neurotransmission (5-HT). Probiotic interventions containing Lactobacillus and Bifidobacterium strains were shown to ameliorate lead-induced depressive-like behaviors, normalize gut microbiota composition, and restore fecal short-chain fatty acid (SCFA) profiles, suggesting a potential preventive and therapeutic strategy against lead-induced neurotoxicity [13,170].

Although longitudinal causal evidence linking lead exposure directly to the onset of depression remains limited, current epidemiological trends and mechanistic studies indicate that lead may contribute to mood dysregulation via oxidative stress, neuroinflammation, immune perturbation, and neurotransmitter disruption, ultimately increasing the risk of depression and other affective disorders.

### 3.2. Arsenic (As)

The International Agency for Research on Cancer (IARC) has classified arsenic (As) as a Group 1 human carcinogen, highlighting its severe threat to human health [171,172]. In addition to its carcinogenicity, arsenic is recognized as a neurotoxicant capable of inducing memory deficits [173]. Epidemiological studies across different countries have suggested a potential association between arsenic exposure and depression. In US adults, urinary arsenic species were significantly correlated with depressive symptoms [174]. Cross-sectional analysis of the NHANES cohort further demonstrated that elevated urinary arsenic levels were associated with a markedly increased risk of adult depressive symptoms. This relationship persisted after adjusting for socioeconomic status, nutritional factors, and inflammatory markers, indicating that environmental arsenic exposure may represent an independent risk factor for depression [175]. Animal studies support the mechanistic link between arsenic exposure and mood dysregulation. Perinatal arsenic exposure in mice disrupts the regulatory interplay between the hypothalamic–pituitary–adrenal (HPA) axis and the dorsal hippocampal serotonergic system, predisposing offspring to depressive-like behaviors [12]. In subchronic arsenic-exposed depression models, arsenic exacerbates depressive phenotypes by inhibiting the BDNF–TrkB signaling pathway in the prefrontal cortex and hippocampus, a pathway critically involved in synaptic plasticity and emotional regulation in depression [176]. In zebrafish, arsenic exposure induces neurobehavioral impairments, including anxiety-, depression-, and autism-like phenotypes, potentially linked to downregulation of immediate-early genes (IEGs) [177]. Mechanistically, arsenic activates immune–inflammatory pathways, particularly through ROS/p38 MAPK-mediated NLRP3 inflammasome activation. NLRP3 activation promotes the release of pro-inflammatory cytokines such as IL-1β and IL-6, exacerbating both mood dysregulation and neural damage, and representing a key driver of neuroinflammation in depression [178]. Arsenic exposure also perturbs neurotransmitter homeostasis, including downregulation of GABA receptor expression, disruption of cholinergic and glutamatergic signaling, and alterations in serotonergic, dopaminergic, and noradrenergic neurotransmission, all of which are classical depression-related pathways [179]. In arsenic-exposed mice, fluoxetine intervention significantly increased hippocampal and prefrontal expression of BDNF, CREB, glucocorticoid receptor (GR), and HDAC2, indicating potential neuroplasticity restoration [180]. Furthermore, studies in in vivo and in vitro models have shown that exposure to heavy metals such as manganese (Mn) can induce Fe^2+^ accumulation, enhanced lipid peroxidation, and depletion of antioxidant defenses, triggering ferroptosis and downregulating miR-125b-2-3p. Upregulation of miR-125b-2-3p alleviates ferroptosis and depressive-like behaviors via targeting transferrin receptor protein 1 (TFR1) [181], suggesting potential convergent pathways shared with arsenic-induced neurotoxicity.

Overall, although most studies remain cross-sectional or preclinical, and heterogeneity exists in exposure dose, duration, and assessment methods, converging evidence from epidemiological and experimental data supports a link between arsenic exposure and depressive phenotypes. The underlying pathogenic mechanisms involve interconnected pathways of oxidative stress, immune–inflammatory activation, mitochondrial dysfunction, and neurotransmitter dysregulation. These findings suggest that environmental arsenic exposure may not only impair cognitive function through chronic neurotoxicity but also contribute to mood dysregulation and depression by activating oxidative and inflammatory cascades. Given arsenic’s widespread environmental presence, its impact on mental health warrants further investigation in large-scale prospective cohorts and mechanistic studies.

## 4. Other Trace Elements

Over the past few decades, the combined effects of survival stress, social environmental changes, and lifestyle transitions have contributed to a continuous rise in the global prevalence of mood disorders, particularly depression, representing a major public health concern. Although the precise etiology of depression remains incompletely understood, accumulating evidence suggests that dynamic imbalances in trace element homeostasis may contribute to its pathogenesis through multi-target mechanisms. Beyond extensively studied elements such as iron, zinc, and selenium, trace elements including copper (Cu), chromium (Cr), magnesium (Mg), and manganese (Mn) also play important roles in neurodevelopment, neurotransmitter metabolism, and emotional regulation. A study investigating metal exposure and neurotransmitter metabolism revealed element-specific associations in urinary metal profiles: chromium was inversely correlated with serotonin levels; cadmium was positively associated with norepinephrine and epinephrine; arsenic correlated positively with tryptophan, serotonin, dopamine, and norepinephrine; iron correlated with elevated homovanillic acid (HVA) and epinephrine; nickel was negatively associated with epinephrine; and zinc showed consistent positive correlations with multiple tryptophan–monoamine pathway metabolites, including kynurenine (KYN), 5-hydroxyindoleacetic acid (5-HIAA), dopamine, HVA, and norepinephrine. In contrast, copper did not exhibit significant associations after adjustment in multi-metal models, collectively suggesting that metal homeostasis imbalance may disrupt monoaminergic neurotransmitter networks through multi-target effects [182]. Copper serves as a cofactor for numerous enzymes and participates in the regulation of depression-related processes. Copper exposure exacerbated depressive-like behaviors in ApoEε4 transgenic mice, potentially via synaptic dysfunction, immune activation, and excessive neuroinflammation, with glial cells playing an important role in the pathogenesis of depression [183,184]. However, population-based studies have reported inconsistent associations between copper status and depression, characterized by marked context dependence and substantial interindividual variability. Surveys conducted among older adults in Australia indicated that serum copper levels were positively correlated with brain-derived neurotrophic factor (BDNF) concentrations and were potentially associated with improved mood status in pregnant women [185,186]. In contrast, among individuals with obesity, higher serum copper levels were associated with an increased risk of depressive symptoms [187]. In addition, epidemiological evidence suggests an inverse association between dietary copper intake and depression risk, although this effect may be partially mediated through copper-induced modulation of the absorption and homeostasis of other trace elements, such as zinc and iron [188]. Collectively, these findings suggest that the influence of copper on depression does not follow a simple linear pattern but may exhibit a potential U-shaped dose–response relationship shaped by copper dosage, individual metabolic status, and the broader context of multi-element interactions. The observed inconsistencies may reflect the bidirectional regulatory effects of copper homeostasis on the nervous system. Within an appropriate physiological range, copper acts as an essential cofactor for multiple enzymes, supporting neuronal metabolism and synaptic plasticity. Conversely, excessive or aberrant copper accumulation may promote reactive oxygen species generation, weaken antioxidant defenses, activate neuroinflammatory pathways, and disrupt neurotransmitter metabolism and synaptic function, thereby impairing neural circuits involved in mood regulation and increasing depression risk [188]. A multi-element dysregulation perspective further helps explain discrepancies across studies. For example, women with relatively low magnesium levels and elevated copper levels have been reported to exhibit a higher likelihood of depressive symptoms [189], suggesting that copper–magnesium homeostasis imbalance may amplify the vulnerability of emotion-regulating networks. Consistent with this notion, cross-sectional analyses based on NHANES data (2009–2018) demonstrated that greater magnesium depletion was significantly associated with increased depression risk [190]. Another population-based study similarly supported a positive association between magnesium depletion and depression prevalence, although causal inference remains limited and requires confirmation in longitudinal studies [191]. At the interventional level, a randomized, double-blind, placebo-controlled trial showed that in patients with depression accompanied by magnesium deficiency, daily supplementation with 500 mg magnesium oxide for at least eight weeks significantly improved serum magnesium levels and alleviated depressive symptoms [192]. Mechanistic studies indicate that magnesium may enhance monoaminergic neurotransmission by modulating serotonergic (5-HT1A/5-HT2A/5-HT2C), noradrenergic (α1/α2), and dopaminergic (D1/D2) receptor activity, and may additionally reduce excitotoxicity through *N*-methyl-D-aspartate (NMDA) receptor antagonism, thereby exerting multi-pathway antidepressant effects [193]. Research on chromium in depression has primarily focused on the interaction between metabolic regulation and emotional behavior [194]. Animal studies have demonstrated that chromium picolinate exerts notable antidepressant- and anxiolytic-like effects in rats exposed to chronic unpredictable mild stress (CUMS), potentially by increasing brain serotonin (5-HT) levels and reducing plasma corticosterone concentrations, thereby mitigating stress-induced affective disturbances [195]. At the metabolic level, chromium may improve insulin utilization efficiency, increase the availability of tryptophan within the central nervous system, and modulate norepinephrine (NA) release, providing a biological basis for its indirect regulation of monoaminergic neurotransmitter systems [196,197]. Given the relatively low direct affinity of chromium for serotonin receptors, the serotonin transporter (SERT), and adrenergic receptors, its putative antidepressant effects are more likely mediated through indirect regulation of metabolic–neural pathways rather than through direct agonistic or antagonistic actions on classical neurotransmitter receptors [198]. Clinical evidence further supports chromium’s potential role in cognitive and emotional regulation. Chromium picolinate supplementation improved cognitive inhibition control and brain function in older adults at risk of neurodegeneration [199]. In patients with type 2 diabetes accompanied by depression or binge-eating symptoms, chromium supplementation may modulate dopamine and serotonin function and regulate prefrontal cortex–ventral tegmental area (PFC–VTA) circuitry, thereby influencing mood and food-related behaviors, with beneficial effects on metabolism-linked mood disorders [200]. Small-sample clinical studies have suggested that chromium supplementation (e.g., chromium picolinate or chromium nicotinate) may enhance the efficacy of conventional antidepressants, although these findings require confirmation in rigorously controlled trials [201]. Further double-blind, placebo-controlled pilot trials reported that in patients with atypical depression, daily supplementation with chromium picolinate (600 μg for 8 weeks) significantly improved specific depressive symptoms, potentially via 5-HT2A receptor downregulation, enhanced insulin sensitivity, and regulation of appetite and carbohydrate craving [202,203]. Subsequent multi-center replication studies further support chromium’s potential to alleviate metabolic-related depressive symptoms through improvements in insulin sensitivity and metabolic behaviors, indicating its potential as an intervention in the depression–metabolic interplay. In contrast, manganese (Mn) exhibits a clear bidirectional role in depression. Epidemiological studies have reported that elevated blood manganese levels are associated with a reduced risk of depression in women, particularly during pregnancy, suggesting that moderate manganese intake may confer emotional protection [204,205,206]. However, excessive manganese exposure can induce neurotoxicity, including neuronal injury and mood disturbances. Mechanistic studies indicate that manganese exposure downregulates miR-125b-2-3p expression and exacerbates iron homeostasis disruption via targeting transferrin receptor protein 1 (TFR1), thereby eliciting anxiety-like behaviors and mood abnormalities [181]. Accordingly, manganese supplementation is not recommended as an intervention for depression in populations with high exposure, such as manganese miners [207]. Overall, although evidence regarding the associations between trace elements such as copper, magnesium, chromium, and manganese and depression remains limited, existing data suggest that disruptions in their homeostasis may contribute to the onset and progression of depression through multiple levels of regulation. Moreover, the homeostatic control of different trace elements is not independent but may be interconnected through metabolic, redox, and neuroendocrine pathways, collectively shaping the pathophysiological milieu associated with depression and facilitating disease development and persistence.

## 5. Network Pharmacology–Based Prediction of Trace Element-Mediated Mechanisms in Depression

Although the preceding sections have systematically reviewed the associations between trace elements and the onset and progression of depression from multiple perspectives, including epidemiological evidence, animal and cellular experiments, and molecular mechanisms, it should be acknowledged that existing studies remain constrained by differences in study design, populations, and analytical approaches. As a result, most investigations have focused on correlations between a single trace element and depressive phenotypes, with mechanistic analyses often centered on individual elements. While this “element-by-element” research paradigm is informative for elucidating the biological functions of specific trace elements, it fails to adequately capture the physiological context in which multiple trace elements coexist and jointly contribute to the development and progression of depression through interactive, synergistic, or antagonistic effects.

Meanwhile, depression is a highly heterogeneous and multisystem-regulated psychiatric disorder, whose pathological basis involves several tightly coupled biological networks, including neurotransmitter metabolism, neuroinflammation, energy metabolism, neuroendocrine regulation, and remodeling of the extracellular microenvironment. Within this context, the traditional “single element–single pathway” framework is insufficient to explain inconsistencies among existing findings and is limited in its ability to elucidate the context dependency, population heterogeneity, and bidirectional dose-related effects of trace elements.

Network pharmacology, as a systems biology–oriented strategy integrating multi-target, multi-pathway, and multi-level information, provides an effective methodological approach to address these limitations. By constructing an integrated network model linking “trace elements–potential targets–signaling pathways–disease phenotypes,” network pharmacology enables the identification of shared regulatory nodes and functional modules through which different trace elements may collectively modulate depressive pathology at a system level. This approach extends beyond the constraints of traditional single-factor analyses and offers a holistic perspective for understanding the complex pathophysiology of depression.

### 5.1. Screening of Trace Element–Related Targets and Construction of the Interaction Network with Depression

Based on the core network pharmacology framework of “multi-component–potential target” analysis, 19 trace elements (Zn, Cr, Fe, Ni, Sn, I, Mn, Si, Pb, Cu, Li, Rb, W, Nb, Se, Mo, Ba, Co, and As) were selected as target elements and used as search keywords. Drug-related and target information were retrieved through an integrated search of the DrugBank database (https://go.drugbank.com/; accessed on 10 September 2024) and the Therapeutic Target Database (TTD; https://db.idrblab.net/ttd/; accessed on 10 September 2024). Potential gene targets that are directly regulated by or specifically bind to these trace elements in the human body were systematically collected, yielding a total of 293 trace element-associated targets after data integration.

To identify depression-related targets, the keywords “Depression,” “Depressive,” and “Depressive Symptoms” were used to search four databases, including GeneCards (https://www.genecards.org/; accessed on 8 January 2025), OMIM (https://www.omim.org/downloads; accessed on 9 January 2025), DrugBank (https://go.drugbank.com/; accessed on 10 January 2025), and TTD (https://db.idrblab.net/ttd/; accessed on 10 January 2025). After data integration and removal of duplicates, a total of 2002 potential core gene targets associated with depression were obtained.

**Figure 3:**
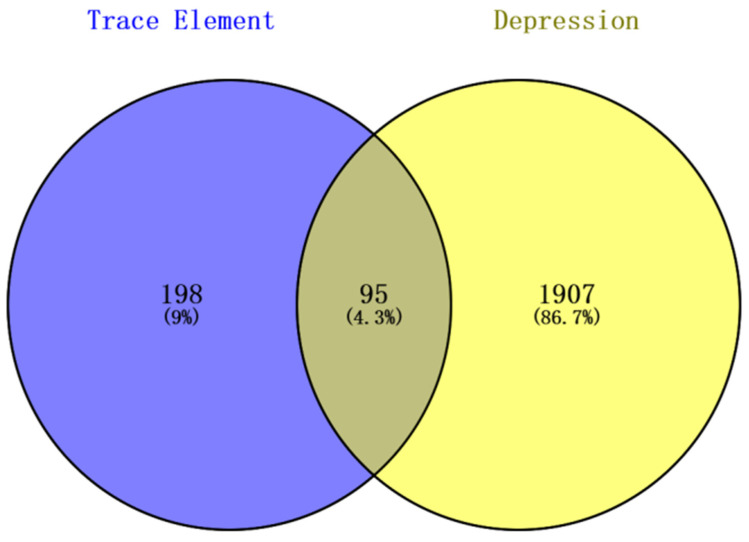
Venn diagram of overlapping targets between trace elements and depression. Note: The blue set represents trace element-related targets (n = 293) and the yellow set represents depression-associated targets (n = 2002). The overlapping region indicates the intersection of trace elements and depression targets (n = 95).

**Figure 4:**
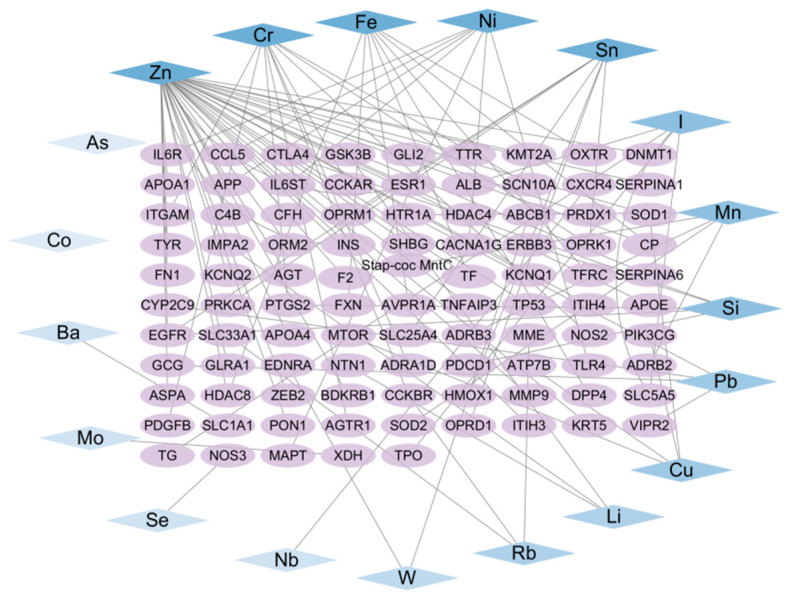
Trace element–potential target network. Note: Light blue diamond-shaped nodes represent trace elements (node color intensity increases with degree value) and pink circular nodes represent potential targets (node labels correspond to gene names).

Subsequently, the Venny 2.1.0 online tool (https://bioinfogp.cnb.csic.es/tools/venny/; accessed on 12 January 2025) was used to perform intersection analysis between the 19 trace element-associated targets and the depression-related targets. A Venn diagram was generated for visualization (Figure 3), identifying 95 overlapping genes jointly regulated by trace elements and depression (defined as core associated targets). The 19 trace elements and the 95 intersecting genes were then imported into Cytoscape 3.10.0 to construct a trace element–potential target interaction network (Figure 4). Network topology analysis was conducted using the built-in Network Analyzer tool, and the Degree value (node connectivity) was applied to evaluate the relative centrality of each trace element within the network. A higher Degree value indicated a greater number of depression-related core targets regulated by a given trace element and thus a stronger central role in the regulatory network. Among all elements, Zn, Cr, and Fe ranked as the top three according to Degree values.

### 5.2. Protein–Protein Interaction Network Analysis and Hub Node Identification

**Figure 5:**
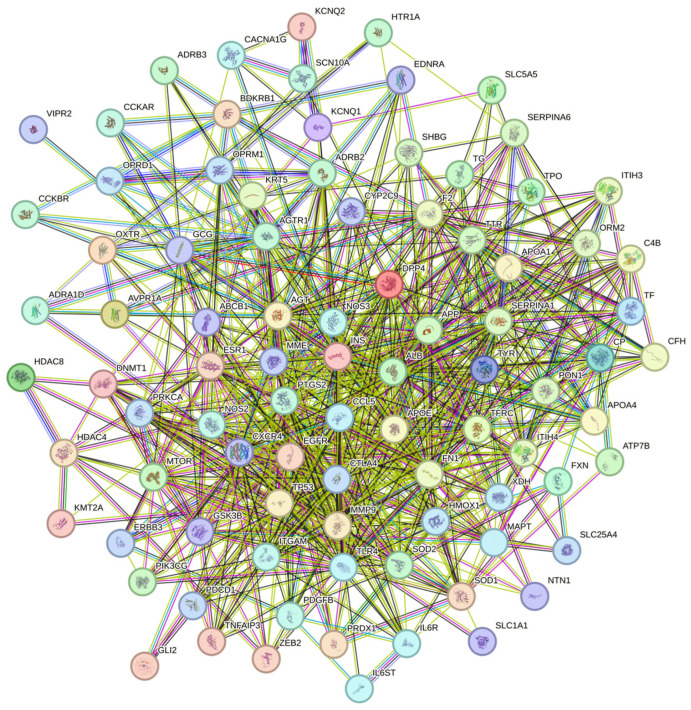
Initial PPI network. Note: The network was constructed using the STRING database. Nodes represent intersection targets between trace elements and depression (labeled by gene names) and edges represent protein–protein interactions.

**Figure 6:**
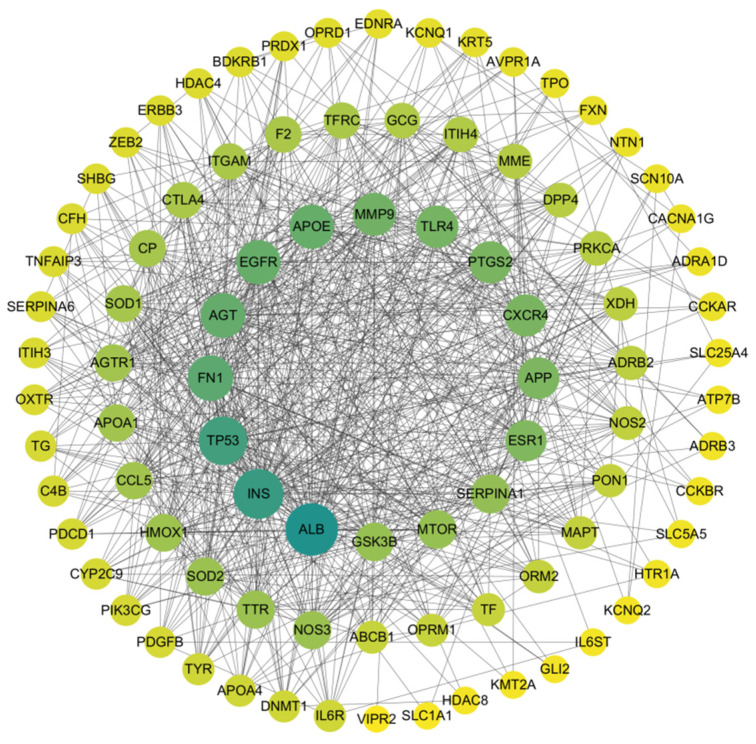
Visualized PPI network. Note: Nodes represent intersection targets between trace elements and depression. Node size and color are proportional to topological centrality (Degree value), with color transitioning from yellow to dark green and size increasing with connectivity, indicating targets with tighter interactions within the network.

The 95 overlapping genes between trace elements and depression were imported into the STRING database (https://string-db.org/; accessed on 13 January 2025). The protein species was restricted to Homo sapiens, and the minimum required interaction score was set to “medium confidence (0.400)” to reduce potential false-positive interactions. Based on these parameters, a protein–protein interaction (PPI) network among the overlapping targets was constructed (Figure 5). The corresponding PPI network data were downloaded in TSV format and further imported into Cytoscape 3.10.0 for visualization and analysis (Figure 6). In the visualized network, node size and color intensity were positively correlated with node degree values; larger nodes with darker colors indicated higher connectivity within the PPI network, reflecting broader protein–protein interactions and greater functional relevance in the network context. Topological analysis of the PPI network was performed using the built-in Network Analyzer tool in Cytoscape. Core targets were identified by ranking nodes according to Degree values (node connectivity). As a result, several targets, including ALB, INS, TP53, FN1, and AGT, were identified as potential anti-depression–related targets of trace elements.

Although these targets have not been definitively defined as core pathogenic molecules of depression, they share common features of high network connectivity and cross-system regulatory capacity, suggesting that they may function as integrative hubs linking trace element homeostasis with biological processes associated with mood disorders. Based on previous studies and the current systems-level analysis, it can be inferred that these targets may act as “signal amplifiers” or “homeostatic regulatory nodes” within trace element–mediated pathological networks of depression.

Among these targets, albumin (ALB), the major carrier protein in plasma, plays an important role in maintaining colloid osmotic pressure, scavenging free radicals, and transporting trace elements and nutrients. These physiological functions are closely related to mechanisms implicated in depression pathology [208,209]. Epidemiological evidence indicates that lower serum albumin levels are significantly and negatively associated with depressive symptoms, an association that has been independently observed across diverse populations, including general adult cohorts, individuals with HIV infection, patients undergoing maintenance hemodialysis, those with chronic liver disease, and middle-aged and older stroke survivors [210,211,212,213,214,215,216].

Serum albumin is the primary binding protein for circulating free zinc, accounting for approximately 60–80% of zinc binding in the bloodstream. Therefore, in the neuropathological context of depression, fluctuations in ALB concentration directly influence zinc kinetic distribution. Reduced albumin-binding capacity may limit the effective delivery of zinc to the synaptic cleft, thereby affecting the modulation of *N*-methyl-D-aspartate (NMDA) receptor function [217,218,219]. Consistently, patients with depression exhibit significantly reduced serum levels of both zinc (Zn) and albumin (ALB), with a positive correlation between the two. The decrease in serum zinc concentration can be partially explained by reduced albumin levels. In addition, depression-associated inflammatory responses, particularly elevated interleukin-6 (IL-6), may promote hepatic sequestration of zinc via metallothioneins, further aggravating peripheral zinc homeostasis imbalance [220].

Supporting this notion, Al-Harthi et al. demonstrated that zinc can influence abnormal protein aggregation by modulating the molecular chaperone function of serum albumin, providing evidence for a potential regulatory role of the “zinc–albumin axis” in maintaining neural functional homeostasis and in the pathogenesis of related neuropsychiatric disorders, including depression [221]. Taken together, serum albumin may serve as a potential biomarker for depression. Its combined roles in antioxidative defense, inflammatory regulation, and maintenance of trace element homeostasis provide a biological basis for understanding the pathological links between trace elements and depression and offer relevant clues for future mechanistic studies and intervention strategies [222].

INS (Insulin/Insulin Signaling) plays an important role in maintaining central nervous system homeostasis by regulating the hypothalamic–pituitary–adrenal (HPA) axis, PI3K/AKT–NMDA/AMPA–ERK–CREB–BDNF signaling cascades, synaptic plasticity, neurogenesis, and autophagy–mitochondrial homeostasis. In addition, insulin signaling suppresses excessive microglial activation and attenuates astrocyte-mediated neuroinflammatory responses, thereby reducing the activation of NF-κB and the NLRP3 inflammasome, as well as the release of pro-inflammatory cytokines such as IL-1β and TNF-α. Insulin also modulates central monoaminergic neurotransmission and neurotrophic factor levels through the gut microbiota–insulin–brain axis, collectively contributing to the maintenance of neural network stability. Impairment of insulin signaling has been shown to induce glial inflammatory imbalance, synaptic dysfunction, and depression-like phenotypes [223,224,225,226,227,228]. Multiple trace elements may exert regulatory effects within this mechanistic network by modulating metabolic and intracellular signaling pathways related to insulin (INS). As described above, chromium, an important component of the glucose tolerance factor, enhances insulin receptor sensitivity and downstream signaling, thereby promoting glucose metabolic homeostasis and improving insulin sensitivity. These effects may stabilize central insulin signaling and indirectly support serotonergic (5-HT) regulation. Magnesium (Mg), as an essential cofactor in insulin signaling pathways, exacerbates insulin resistance when deficient, which may further impair insulin-mediated mood regulation [229,230]. Zinc (Zn) is closely involved in insulin secretion and signal transduction; zinc supplementation has been shown to improve insulin sensitivity, whereas zinc deficiency is also associated with neurotransmitter imbalance and depressive symptoms [114,115,231].

P53 functions as a stress-responsive transcription factor that can be activated under conditions of oxidative damage, metabolic dysregulation, and chronic inflammation, thereby influencing neuronal survival, mitochondrial function, energy metabolism, and synaptic plasticity. Accumulating evidence indicates that such stress responses in the central nervous system are closely associated with affective disturbances. Depression-like behaviors have been linked to neuroinflammation, oxidative stress, and dysregulated apoptosis, with aberrant activation or dysfunction of p53 potentially serving as an important molecular link among these processes. Animal models and clinical transcriptomic analyses further support this association. In inflammation-related depression, peripheral blood transcriptome profiles from individuals with obesity have shown marked alterations in TP53-, glucocorticoid receptor (GR)-, and NF-κB–related pathways. Excessive GR activation may amplify inflammatory responses and apoptotic signaling, whereas weight-loss interventions can alleviate both inflammation and depressive symptoms. These findings suggest that depression may be mediated, at least in part, through an inflammation–glucocorticoid–apoptosis signaling axis, which may serve as a molecular feature of inflammation-associated major depressive disorder [232]. Functional studies have further demonstrated that in chronic stress– or corticosterone-induced depression-like mouse models, p53 promotes behavioral abnormalities by inducing oxidative stress and neuroinflammatory signaling pathways (e.g., the DDIT4–NF-κB axis). Inhibition of p53 activity, such as through pifithrin-α treatment, partially alleviates anxiety- and depression-like behaviors while restoring dendritic structure and antioxidant capacity. These findings suggest that p53-mediated signaling pathways may represent potential therapeutic targets in depression pathology [233,234]. Within this pathological framework, multiple trace elements influence p53 function and downstream neurobiological processes by modulating cellular stress responses, redox homeostasis, metal-dependent enzyme activity, and intracellular signaling. On the one hand, metal ions such as zinc (Zn) and copper (Cu) directly affect the structural stability and DNA-binding capacity of p53. Zinc regulates p53 structural domains and thereby alters transcriptional control of target genes, whereas excessive copper may interfere with zinc-binding sites within p53, leading to abnormal protein folding and functional impairment. This mechanism suggests that metal homeostasis disturbances may influence p53-regulated cell fate decisions [235,236,237]. On the other hand, trace element imbalance may indirectly regulate p53 activity by promoting oxidative stress and inflammatory signaling. Elements such as Zn, selenium (Se), and magnesium (Mg), which participate in antioxidant defense systems, have been inversely correlated with inflammatory markers and oxidative damage indicators in both depressive populations and animal models. Deficiency of these elements may enhance oxidative stress, thereby facilitating excessive p53 activation and triggering neuroapoptosis and inflammatory responses, ultimately creating a molecular environment conducive to depressive phenotypes [238,239]. In contrast, environmental exposure to heavy metals such as lead (Pb), arsenic (As), and cadmium has been reported to induce oxidative damage and inflammatory states, which can activate p53 and downstream stress responses, potentially contributing to depression-like pathology [240,241,242,243]. Although the precise molecular details of direct trace element–p53 interactions remain under investigation, a common theme is their convergence on oxidative/antioxidative balance, inflammation, and cell death pathways within p53-dependent signaling networks.

Fibronectin 1 (FN1) is a major extracellular matrix (ECM) glycoprotein that regulates cellular behavior, matrix remodeling, and local stress responses through interactions with integrin receptors and other ECM components, such as collagen and heparin. Proteomic and brain ECM studies have linked alterations in FN1 or its homologs under psychiatric conditions to disturbances in emotional regulation, suggesting potential interactions between ECM remodeling and depression-related protein networks [244,245]. Although direct mechanistic evidence linking FN1 to depression remains limited, its functional roles in other neurodegenerative and stress-related conditions provide relevant analogies. For example, in Alzheimer’s disease models, changes in FN1 expression have been associated with blood–brain barrier ECM composition, microglial activation, and neuroinflammatory responses. Functional loss-of-FN1 studies have demonstrated attenuation of inflammatory glial responses, indicating a close relationship between ECM composition and neuroinflammation—mechanisms that are also repeatedly implicated in depression pathology [246,247].

Angiotensinogen (AGT), the precursor substrate of the renin–angiotensin system (RAS), exerts key endocrine and paracrine functions in both systemic circulation and local tissues. Its cleavage product, angiotensin II (Ang II), mediates a broad range of physiological and pathological effects primarily through activation of the AT1 receptor [248]. Multiple reviews have demonstrated that elevated RAS activity promotes depressive and anxiety-related symptoms by inducing oxidative stress and inflammatory responses. Moreover, angiotensin-converting enzyme inhibitors and Ang II receptor blockers (ARBs) have exhibited anti-inflammatory and antidepressant-like effects in both clinical studies and animal models, providing a theoretical basis for targeting RAS in psychiatric disorders [249,250,251]. As discussed above, deficiencies in trace elements such as Zn, Mg, and Se commonly observed in depression are closely associated with systemic inflammation and oxidative stress, which are also key downstream consequences of RAS overactivation. Although direct experimental evidence linking specific trace elements to AGT/Ang II signaling remains limited, the shared involvement of RAS, inflammation, oxidative stress, and neurotransmitter regulation suggests that optimization of trace element status may represent a metabolically oriented strategy for modulating depression-related pathways, warranting further experimental investigation.

Taken together, the network-based results of this study indicate that trace element–related targets are not confined to a single depression-specific signaling pathway. Instead, they are predominantly enriched in system-level pathways involving inflammatory and immune regulation, energy metabolism, neural plasticity, HPA axis modulation, and vascular and extracellular matrix remodeling. This pattern is consistent with the mechanistic evidence discussed above and further supports the notion that trace elements influence depression primarily through homeostasis-driven, network-level pathological remodeling rather than isolated molecular effects.

### 5.3. GO Functional Enrichment and KEGG Pathway Analysis

**Figure 7:**
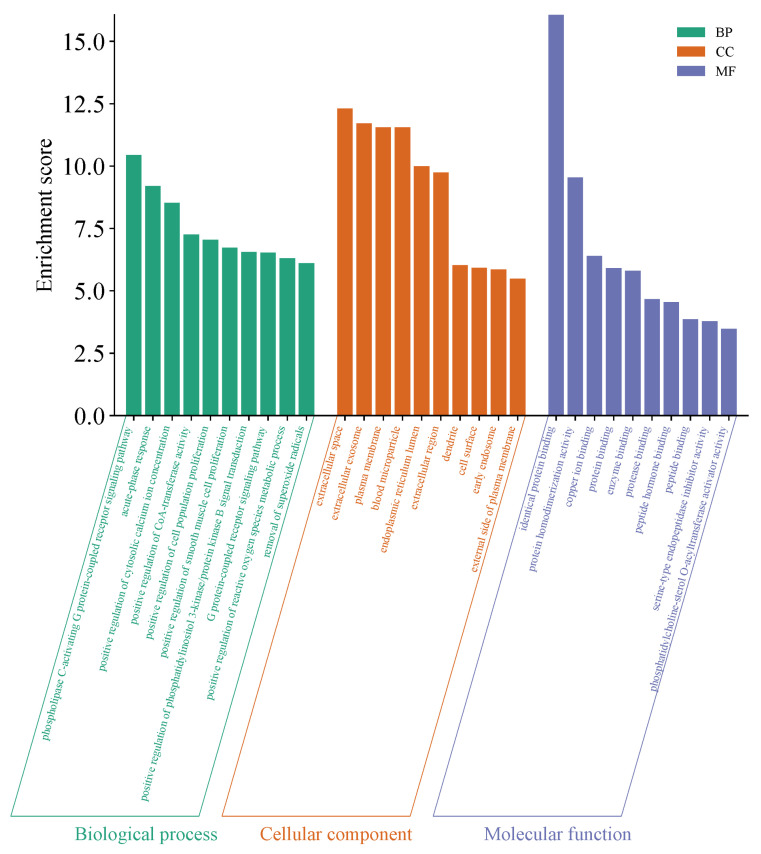
Top 10 enriched GO terms for Biological Process (BP), Cellular Component (CC), and Molecular Function (MF). Note: The top 10 enriched terms in each category are shown: BP (green), CC (orange), and MF (purple). The y-axis represents the Enrichment Score, with higher values indicating a stronger association between the term and the targets.

The potential therapeutic targets of trace elements for depression were imported into the DAVID database (https://david.ncifcrf.gov/; accessed on 18 January 2025) for Gene Ontology (GO) functional enrichment analysis and Kyoto Encyclopedia of Genes and Genomes (KEGG) pathway analysis. The gene identifier was set as “OFFICIAL_GENE_SYMBOL,” and the species was restricted to Homo sapiens to ensure the specificity and accuracy of functional annotations. GO enrichment analysis was conducted across three categories: Biological Process (BP), Cellular Component (CC), and Molecular Function (MF). The top 10 enriched terms for BP, CC, and MF, as well as the top 20 enriched KEGG pathways, were selected for visualization using the bioinformatics online platform. A total of 448 GO terms were enriched, including 319 Biological Process (BP) terms, 59 Cellular Component (CC) terms, and 70 Molecular Function (MF) terms. The top 10 terms in each GO category were visualized using the bioinformatics online platform (Figure 7), with the “enrichment score” serving as the primary evaluation metric; higher scores indicated stronger associations between the functional terms and the target gene set.

At the level of biological processes, the enriched terms were mainly related to the phospholipase C–activating G protein–coupled receptor (GPCR) signaling pathway, acute-phase response, and positive regulation of cytosolic calcium ion concentration. These findings suggest that GPCR-mediated signal transduction, inflammatory responses, and calcium homeostasis regulation may represent important biological processes through which trace elements participate in the modulation of depression-related pathology. Mechanistically, GPCR signaling cascades can activate the Gα_q/phospholipase C (PLC) pathway, leading to Ca^2+^ release from the endoplasmic reticulum and subsequent regulation of intracellular second messenger systems, as well as the expression of stress-responsive genes such as c-fos [252]. Dysregulation of this signaling axis may result in neuronal calcium homeostasis imbalance and altered calcium signaling patterns, which have been implicated as important mechanisms underlying chronic stress-induced impairments in neural plasticity and emotional behavior [253,254,255]. Calcium signaling pathways are closely interconnected with oxidative stress and mitochondrial function. Excessive reactive oxygen species (ROS) generated under conditions of iron homeostasis imbalance can activate calcium-dependent phospholipase A_2_, promoting the release of membrane polyunsaturated fatty acids and amplifying lipid peroxidation processes. In iron overload-driven depression models, this mechanism contributes to a vicious cycle involving oxidative stress, calcium signaling dysregulation, and neuroinflammation. These findings highlight the complex interactions between calcium homeostasis and other trace elements, such as iron, zinc, and magnesium, within the depression-related pathological network [256,257].

At the cellular component (CC) level, the enriched targets were mainly distributed in the extracellular space, extracellular exosomes, and plasma membrane. This spatial distribution is closely associated with the regulation of the neuroinflammatory microenvironment and extracellular signal transmission. Previous studies have demonstrated that dysregulation of inflammatory mediators, exosome-mediated signaling, and membrane receptor function constitutes an important component of neural circuit dysfunction in depression. Accordingly, the present findings provide spatial-level support for the involvement of trace elements in the regulation of neuroinflammation and extracellular signaling homeostasis [258,259]. At the molecular function (MF) level, the enriched terms were primarily related to identical protein binding, protein homodimerization activity, and copper ion binding. These molecular functions are fundamental for maintaining the stability of neurotransmitter transporters (e.g., NET and VMAT), receptor complex assembly, and signal transduction processes. Protein homodimerization is essential for the activation and functional regulation of multiple G protein–coupled receptors (GPCRs), receptor tyrosine kinases (RTKs), and neurotransmitter transporters, and its disruption may impair the homeostatic regulation of glutamatergic and GABAergic neurotransmission. Collectively, these molecular function characteristics provide a plausible molecular basis for the neuromodulatory roles of trace elements (such as Zn, Mg, Fe, and Cu) through their regulation of protein–protein interaction interfaces [260,261]. Specifically, Zn and Mg act as structural or functional cofactors for numerous proteins, stabilizing protein complexes and modulating the conformational states of Ca^2+^-dependent receptors and ion channels [262,263]. In contrast, Fe and Cu indirectly regulate protein binding properties and signaling efficiency by influencing the activity of key enzymes involved in neurotransmitter synthesis (such as tyrosine hydroxylase and dopamine β-hydroxylase) as well as redox-sensitive structural domains, thereby participating in depression-related pathological processes across multiple regulatory layers [82,264].

**Figure 8:**
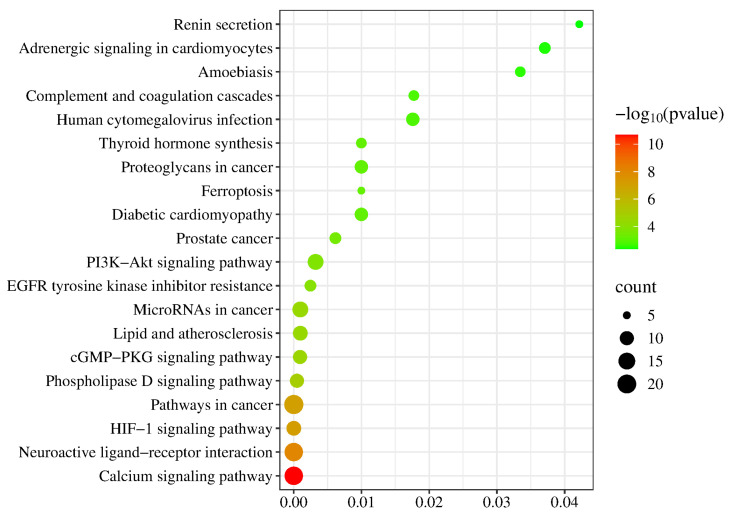
Bubble plot of the top 20 enriched KEGG pathways. Note: The x-axis represents the enrichment factor and the y-axis denotes the pathway name. Bubble color corresponds to −log10(*p*-value), with redder colors indicating higher enrichment significance. Bubble size represents the number of targets within the pathway (count), with larger bubbles indicating more targets.

KEGG pathway enrichment analysis using the DAVID database identified a total of 72 significantly enriched pathways, of which the top 20 were visualized using the MicrobiomeAnalyst (Weishengxin) online platform (Figure 8). In this analysis, the *p* value represented the statistical reliability of pathway enrichment (*p* < 0.05 indicated statistical significance), while −log_10_(*p* value) reflected a logarithmic transformation of the *p* value, whereby higher values corresponded to greater statistical significance and a lower risk of false-positive enrichment. The count value denoted the number of core targets involved in a given pathway, with higher counts indicating a more prominent functional position within the network. Among all enriched pathways, the calcium signaling pathway exhibited the highest level of significance, as indicated by the highest −log_10_(*p* value) and a relatively large number of associated targets, with the bubble color approaching red. This finding suggests that calcium signaling may occupy a central hub position in trace element-mediated regulation of depression-related pathological processes. Previous studies have shown that modulation of calcium signaling can rapidly alleviate depression-like behaviors by regulating glutamatergic activity, postsynaptic Ca^2+^ dynamics, and synaptic plasticity. These effects exhibit functional similarities to the rapid antidepressant actions mediated by ketamine, thereby further supporting the potential importance of trace elements acting in concert with this pathway in depression treatment [265]. The second most significantly enriched pathway was neuroactive ligand–receptor interaction, which encompassed a wide range of central nervous system ligands and their corresponding receptors, including neuroactive steroids, γ-aminobutyric acid (GABA), and serotonin (5-hydroxytryptamine, 5-HT). This pathway plays a key role in regulating neurotransmitter transmission, excitatory–inhibitory balance, and neuroinflammatory homeostasis through high-affinity ligand–receptor interactions and constitutes an important molecular basis for emotional regulation and antidepressant treatment responses [266]. Disruption of trace element homeostasis may indirectly influence depressive pathology by affecting the stability and functional state of ligand–receptor signaling complexes within neurotransmitter regulatory networks [267,268]. In addition, the hypoxia-inducible factor 1 (HIF-1) signaling pathway was also significantly enriched. This pathway has been implicated in the pathogenesis of depression through its involvement in mitochondrial dysfunction, metabolic reprogramming, and astrocyte apoptosis. Existing evidence indicates that environmental toxic elements such as lead can interact with HIF-1α, thereby amplifying hypoxic and oxidative stress signaling, exacerbating neuronal injury, and increasing the risk of mood disturbances [169]. These findings provide a potential molecular link between exposure-related trace elements and depression.

Taken together, the GO and KEGG enrichment results demonstrate a high degree of concordance at both functional and spatial levels, indicating that the identified targets are not randomly distributed but instead converge on key pathways related to neurotransmission regulation, neuroinflammatory responses, stress adaptation, and energy metabolism. From a systems perspective, these pathways collectively outline a core functional network through which trace elements may participate in the pathological regulation of depression. It should be emphasized, however, that pathway enrichment analyses primarily reflect potential regulatory associations and functional networks, and their precise biological significance requires integration with existing experimental evidence as well as further validation through mechanistic studies and clinical investigations.

### 5.4. Advantages and Limitations of Network Pharmacology Analysis

In this study, a network pharmacology approach was employed to construct an integrated “trace element–target–depression” network from a systems biology perspective, providing a complementary framework for elucidating how multiple trace elements may cooperatively contribute to the onset and progression of depression through multi-target and multi-pathway regulation. By mapping literature-derived candidate targets onto a protein–protein interaction (PPI) network, this strategy facilitates the identification of functionally convergent regulatory pathways and network hub nodes, thereby revealing, at a global level, potential patterns through which trace element homeostasis imbalance may induce network-wide perturbations in emotion-regulatory systems. It should be emphasized that the network pharmacology analyses performed in this study are primarily hypothesis-generating in nature, aiming to provide structured mechanistic clues rather than to infer causality or validate specific molecular mechanisms directly.

From a methodological standpoint, network pharmacology analyses rely heavily on the integration of databases such as GeneCards, DrugBank, and the Therapeutic Target Database (TTD). Consequently, the results are inevitably influenced by limitations in database annotation completeness, prediction algorithm bias, and background noise. Therefore, the key targets and enriched pathways identified through network analysis should not be equated with definitive pathogenic mechanisms and must be further confirmed through experimental studies and clinical validation [269]. Moreover, hub genes identified via PPI network analysis are more likely to function as central regulatory nodes within systemic biological networks rather than as depression-specific biomarkers. In this context, these hub genes are interpreted as functional nodes within trace element–regulated depression-related networks, highlighting their integrative systems-level significance rather than disease-specific diagnostic value.

Another aspect requiring cautious interpretation is that the “trace element–target” associations established in databases are highly dependent on factors such as dosage, chemical form, bioavailability, and tissue-specific distribution. These critical variables are difficult to adequately capture within current network models [270,271]. Accordingly, the hub genes and pathways identified in this study are incorporated into a conceptual framework of “trace element homeostasis–multi-pathway regulation–depressive phenotype heterogeneity,” aiming to characterize system-level network disturbances induced by trace element imbalance rather than to designate explicit causal targets.

Overall, the primary value of network pharmacology analysis lies in theoretical integration and hypothesis generation. By identifying shared pathways and network hub nodes, this approach contributes to a deeper understanding of the biological complexity of depression and provides directional guidance for subsequent mechanistic validation, multi-omics integration, and longitudinal investigations.

## 6. Discussion and Conclusions

**Figure 9:**
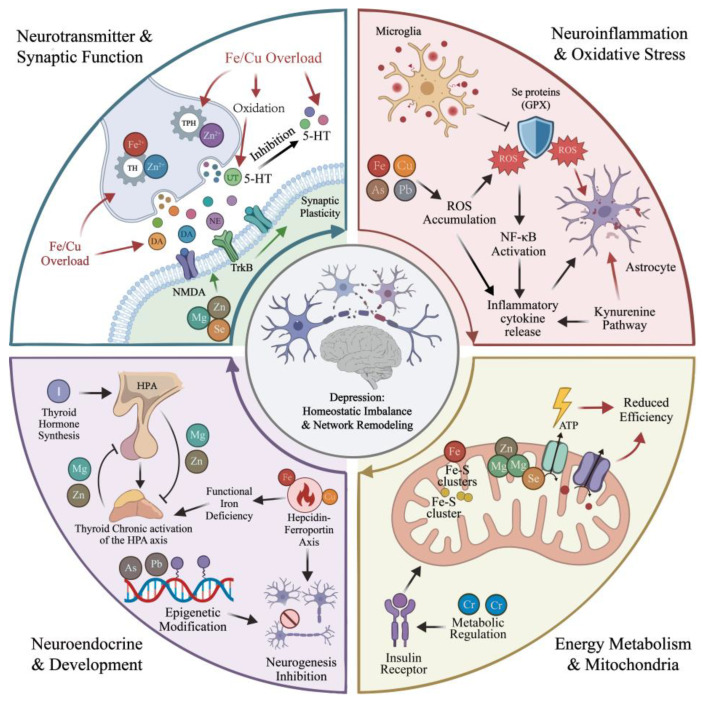
Schematic illustration of the mechanisms linking trace elements to the pathogenesis of depression. Solid arrows indicate direct regulatory or causal relationships supported by relatively robust experimental evidence; T-shaped terminal lines indicate inhibitory or negative regulatory effects.

This work systematically integrates evidence linking multiple trace elements—including zinc, selenium, iron, iodine, chromium, and manganese—to the onset and progression of depression, encompassing epidemiological observations, animal experiments, clinical interventions, and molecular mechanisms. Collectively, existing studies indicate that essential trace elements such as iron, zinc, selenium, chromium, iodine, magnesium, and copper, as well as environmentally toxic elements including arsenic and lead, participate in depressive pathology through multi-level and cross-system biological mechanisms. A unifying feature of these effects is network-level pathological remodeling driven by disturbances in trace element homeostasis (Figure 9).

At the neurotransmitter level, iron and zinc act as indispensable cofactors for key enzymes such as tyrosine hydroxylase, tryptophan hydroxylase, and aromatic L-amino acid decarboxylase. Deficiency of these elements can directly suppress the synthesis of dopamine (DA), norepinephrine (NE), and serotonin (5-HT), resulting in reduced central monoaminergic signaling. Conversely, iron or copper overload may exacerbate neurotransmitter imbalance by promoting monoamine auto-oxidation and inhibiting enzymatic activity. In parallel, zinc, magnesium, and selenium modulate NMDA and GABA receptor function, 5-HT_1*A*_/5-HT_2*A*_ signaling, and the BDNF–TrkB pathway, thereby exerting profound effects on synaptic plasticity and the stability of emotion-regulatory networks.

With respect to neuroinflammation and oxidative stress, selenium-dependent selenoproteins (e.g., GPx and TrxR) constitute a critical defense system maintaining cerebral redox homeostasis. Insufficient selenium availability can amplify reactive oxygen species (ROS) accumulation and activate inflammatory cascades such as NF-κB and the NLRP3 inflammasome. In contrast, excess iron, copper, arsenic, and lead markedly enhance oxidative stress through Fenton chemistry or mitochondrial toxicity, thereby promoting pro-inflammatory microglial polarization and impairing the neurotrophic support provided by astrocytes. Sustained low-grade inflammation further accelerates depressive phenotypes via kynurenine pathway shunting, BDNF downregulation, and impaired neurogenesis.

At the level of energy metabolism and mitochondrial function, iron is central to Fe–S cluster and heme biosynthesis, while zinc, magnesium, and selenium contribute to the structural stability of oxidative phosphorylation complexes and antioxidant protection. Dysregulation of multiple trace elements can synergistically compromise respiratory chain efficiency and ATP production, particularly affecting emotion-related brain regions such as the hippocampus, prefrontal cortex, and nucleus accumbens. These deficits weaken synaptic plasticity and stress adaptability. Chromium, by enhancing insulin sensitivity and modulating central energy metabolism and tryptophan availability, provides a mechanistic link between metabolic dysfunction and depression, highlighting a metal-dependent basis for depression–metabolism comorbidity.

From neuroendocrine and developmental perspectives, iodine deficiency disrupts thyroid hormone synthesis, thereby interfering with brain development, synaptic maturation, and emotional homeostasis in adulthood. Magnesium and zinc modulate negative feedback regulation of the hypothalamic–pituitary–adrenal (HPA) axis, whereas iron and copper, together with inflammation-driven dysregulation of the hepcidin–ferroportin axis, may induce a state of “functional neuronal iron deficiency,” sustaining chronic HPA axis activation. Environmental toxicants such as arsenic and lead further increase depression susceptibility through epigenetic modifications, impaired neurogenesis, and disrupted neural network remodeling, with particularly pronounced effects during early development and in highly exposed populations.

Building on this evidence, the present study employed a network pharmacology framework to predict key trace element–associated targets (e.g., ALB, INS, TP53, FN1, and AGT) and related signaling pathways, including neuroinflammatory signaling, calcium signaling, neuroactive ligand–receptor interactions, and stress-response pathways. These findings provide system-level hypotheses for subsequent mechanistic validation; however, their causal relevance must be confirmed through targeted experimental and clinical studies.

Importantly, current evidence does not support the indiscriminate or empirical use of non-specific trace element supplementation as a general intervention for depression in the absence of rigorous deficiency assessment and professional clinical guidance. Modulation of trace element homeostasis is more likely to be beneficial in specific susceptible subpopulations, and any intervention should be implemented cautiously under medical supervision, with careful consideration of overall metabolic status, endocrine background, and comorbid conditions.

In summary, trace elements do not influence depression through isolated targets or single pathways but are embedded within a highly coupled regulatory network encompassing neurotransmission, inflammation, energy metabolism, and neuroendocrine control. Their biological effects exhibit pronounced dose dependence, temporal sensitivity, and population heterogeneity. This systems-level perspective deepens our understanding of the complex pathophysiology of depression and provides a theoretical foundation for the development of precision nutrition strategies and individualized adjunctive therapeutic approaches. Future studies should integrate multi-omics data, longitudinal population-based investigations, and mechanistic validation experiments to systematically elucidate the causal relationships between trace element homeostasis dysregulation and distinct depression subtypes, thereby facilitating the translation of network-based predictions into clinically actionable evidence.

## Data Availability

No new data were created or analyzed in this study. Data sharing is not applicable to this article.
